# A Meningococcal Outer Membrane Vesicle Vaccine Incorporating Genetically Attenuated Endotoxin Dissociates Inflammation from Immunogenicity

**DOI:** 10.3389/fimmu.2016.00562

**Published:** 2016-12-08

**Authors:** David J. Dowling, Holly Sanders, Wing Ki Cheng, Sweta Joshi, Spencer Brightman, Ilana Bergelson, Carlo Pietrasanta, Simon D. van Haren, Sandra van Amsterdam, Jeffrey Fernandez, Germie P. J. M. van den Dobbelsteen, Ofer Levy

**Affiliations:** ^1^Department of Medicine, Division of Infectious Diseases, Boston Children’s Hospital, Boston, MA, USA; ^2^Harvard Medical School, Boston, MA, USA; ^3^Janssen Vaccines and Prevention B.V., Leiden, Netherlands; ^4^Precision Vaccine Program, Division of Infectious Diseases, Boston Children’s Hospital, Boston, MA, USA; ^5^Neonatal Intensive Care Unit, Department of Clinical Sciences and Community Health, Fondazione IRCCS Ca’ Granda Ospedale Maggiore Policlinico, University of Milan, Milan, Italy; ^6^Janssen Research and Development, LLC, Spring House, PA, USA

**Keywords:** group B meningococci, outer membrane vesicles, vaccine, newborn, dendritic cells

## Abstract

**Background:**

Group B *Neisseria meningitidis*, an endotoxin-producing Gram-negative bacterium, causes the highest incidence of group B meningococcus (MenB) disease in the first year of life. The Bexsero vaccine is indicated in Europe from 8 weeks of age. Endotoxin components of outer membrane vesicles (OMVs) or soluble lipopolysaccharide (LPS) represent a potential source of inflammation and residual reactogenicity. The purpose of this study was to compare novel candidate MenB vaccine formulations with licensed vaccines, including Bexsero, using age-specific human *in vitro* culture systems.

**Methods:**

OMVs from wild type- and inactivated *lpxL1* gene mutant-*N. meningitidis* strains were characterized in human neonatal and adult *in vitro* whole blood assays and dendritic cell (DC) arrays. OMVs were benchmarked against licensed vaccines, including Bexsero and whole cell pertussis formulations, with respect to Th-polarizing cytokine and prostaglandin E2 production, as well as cell surface activation markers (HLA-DR, CD86, and CCR7). OMV immunogenicity was assessed in mice.

**Results:**

Δ*lpxLI* native OMVs (nOMVs) demonstrated significantly less cytokine induction in human blood and DCs than Bexsero and most of the other pediatric vaccines (e.g., PedvaxHib, EasyFive, and bacillus Calmette–Guérin) tested. Despite a much lower inflammatory profile *in vitro* than Bexsero, Δ*lpxLI* nOMVs still had moderate DC maturing ability and induced robust anti-*N. meningitidis* antibody responses after murine immunization.

**Conclusion:**

A meningococcal vaccine comprised of attenuated LPS-based OMVs with a limited inflammatory profile *in vitro* induces robust antigen-specific immunogenicity *in vivo*.

## Introduction

Human newborns and infants suffer a high frequency of infection compared to older children and adults (1), in part due to distinct early life immunity with impaired host defense (2). Early life immunization is desirable, but vaccine-induced responses of newborns and young infants demonstrate slow initiation, low immunogenicity, and reduced persistence of functional antibodies and cell-mediated responses (2). Although the majority of global immunization schedules are focused on the pediatric age group, development of early life vaccines has been hampered by this distinct immunity and an *ad hoc* approach to developing vaccines, relying on adult-derived results that may inaccurately predict infant responses (3).

*Neisseria meningitidis* (meningococcus) is a Gram-negative, endotoxin-producing organism that is a normal commensal of the human nasopharynx and is an important cause of invasive bacterial infection in children worldwide (4). Recent immunization programs with capsular-polysaccharide vaccines have dramatically reduced the incidence of serogroup C and A meningococcal disease in North America, Europe, and Africa (5). However, meningitis and septicemia caused by serogroup B meningococci remain a major health concern in young children, as similar capsule-based vaccines cannot be developed against this serogroup (6). Outer membrane vesicle (OMV)-based vaccines have historically been used, with some success, to control outbreaks of disease caused by serogroup B meningococci. OMVs are produced by the blebbing of membranes of clinical-derived live Gram-negative bacteria during *in vitro* growth and are useful vaccine components, as immuno-stimulatory membrane components [lipids, proteins, lipopolysaccharide (LPS), etc.] from meningococci are represented (7). As OMV yield from culture alone is too low for vaccine production, detergent extraction is used to force vesiculation and increase yield and has the added advantage of reducing LPS content to prevent overt reactogenicity. Novartis’ 4-component aluminum hydroxide (Alum)-adjuvanted group B meningococcus (MenB) vaccine (4CMenB, Trade name: Bexsero) has been licensed by both the European Medicines Agency and the U.S. Food and Drug Agency and comprised OMVs and three recombinant immunogenic *N. meningitidis* proteins identified by reverse vaccinology (8). Another meningococcal serogroup B vaccine, Trumenba, contains two recombinant proteins with no OMV component, is approved in the U.S. for use in individuals 10 through 25 years of age, but lacks the potentially broader antigen repertoire inherent to OMVs.

The currently licensed meningococcal vaccines have the potential to reduce mortality and morbidity associated with MenB infections, but do have some limitations. Although detergent extraction of OMVs removes the majority of the LPS, the remaining endotoxin, particularly the soluble LPS, may still result in residual reactogenicity, necessitating the use of the Alum to ameliorate excess toxicity (9). Even so, immunization with the OMV-containing Bexsero may correlate with rates of reactogenicity (e.g., fever ≥38°C, tenderness at injection site) as common as 10% in infants less than 1 years of age (10), and severe reactogenicity is reported in some cases (11). Bexsero may also enhance reactogenicity when given together with other vaccines (12), prompting some primary care physicians to prescribe anti-inflammatory agents (13) to prevent potential reactions. Furthermore, as conventional assays for preclinical and release testing of vaccines, such as the rabbit pyrogenicity test and *Limulus* amebocyte lysate (LAL) assay, were developed for vaccines containing no or negligible amounts of endotoxin, predicting the reactogenicity of LPS-containing OMV vaccines is difficult and requires more sophisticated models (14).

To overcome the potential reactogenicity of wild-type (WT) OMV-based vaccines while maintaining the inherent adjuvant activity of vesicles, a vaccine has previously been developed using a *N. meningitidis* strain with genetically attenuated endotoxin (15). This enabled the use of detergent-free manufacturing processes to produce native OMVs (nOMVs) (16). To assess the potential of such OMV vaccines, we reasoned that it would be important to better understand their interactions with human leukocytes, including dendritic cells (DCs). DCs are professional antigen-presenting cells (APCs) that play a vital role in shaping adaptive immunity. DC maturation begins when endogenous or exogenous danger molecules are recognized by pattern recognition receptors [e.g., *Toll*-like receptors (TLRs)] triggering upregulation of costimulatory molecules and production of immune polarizing cytokines (17). Of note, in addition to interactions *via* TLR4, *N. meningitidis* LPS also directly interacts with DCs through the C-type lectin receptor DC-SIGN and modulates their function (18).

In this study, we benchmarked novel candidate MenB vaccine formulations against licensed vaccines, including Bexsero, using physiologically relevant human neonatal and adult whole blood (WBA) and monocyte-derived dendritic cell (MoDC) *in vitro* culture systems, both of which employ age-specific autologous plasma, a rich source of age-specific immunomodulatory molecules (2, 19). We assessed vaccine-induced production of Th-polarizing cytokines, upregulation of surface activation markers (20), and production of potential vaccine reactogenicity biomarkers such as IL-1β and prostaglandin E2 (PGE_2_) (3, 21, 22). We found that a meningococcal vaccine comprised of attenuated LPS-based OMVs with a relatively low inflammatory profile toward human leukocytes *in vitro* induced robust antigen-specific immunogenicity *in vivo*. Our observations, may inform translational development of MenB vaccines in high-risk populations, such as newborns and infants.

## Materials and Methods

### Ethics Statement

Peripheral blood samples were collected after written informed consent from healthy adult volunteers with approval from the Ethics Committee of Boston Children’s Hospital, Boston, MA, USA (X07-05-0223). Non-identifiable cord blood samples were collected immediately after elective cesarean section delivery (epidural anesthesia) of the placenta. Births to HIV-positive or febrile mothers were excluded. Human experimentation guidelines of the U.S. Department of Health and Human Services, the Brigham and Women’s Hospital, Beth Israel Medical Center, and Boston Children’s Hospital were observed, with approval from the Ethics Committee of The Brigham and Women’s Hospital, Boston, MA, USA (protocol number 2000-P-000117) and Beth Israel Deaconess Medical Center Boston, MA, USA (2011P-000118). Mice were obtained from Envigo (Indianapolis, IN, USA), and studies were approved by the Janssen Research and Development, LLC Institutional Animal Care and Use Committee.

### Human Blood Processing and *In Vitro* Stimulation

Human blood was anti-coagulated with 20 U/ml pyrogen-free sodium heparin (American Pharmaceutical Partners, Inc., Schaumberg, IL, USA). All blood products were kept at room temperature and processed within 4 h from collection. Whole blood (WB) was mixed 1:1 with pre-warmed (37°C) RPMI 1640 medium (Invitrogen, Carlsbad, CA, USA) and 180 μl plated in a 96-well U-bottom plate (Becton Dickinson, Franklin Lakes, NJ, USA) containing 20 μl of 10× freshly prepared treatment, as described previously (23).

### Human MoDCs

Blood was layered onto Ficoll-Hypaque gradient (GE Healthcare, Waukesha, WI, USA) to collect cord blood mononuclear cells (CBMCs) or peripheral blood mononuclear cells (PBMCs). Monocytes were isolated by positive CD14 selection with magnetic microbeads (Miltenyi Biotec, Auburn, CA, USA). Preparations were routinely >95% pure as assessed by flow cytometry (23–26). Monocytes were cultured in tissue culture dishes at 0.4 × 10^6^ cells/ml in RPMI media containing fresh 10% autologous platelet-poor plasma, supplemented with recombinant human (rh) IL-4 (50 ng/ml) and rh granulocyte-macrophage colony-stimulating factor (rhGM-CSF) (100 ng/ml) (R&D Systems, Minneapolis, MN, USA) with one replenishment of fresh media/cytokines at day 3. After 5–6 days, immature MoDCs were handled, as described previously (23, 25).

### Vaccines, TLR Agonists, and Multi-Analyte Assays

Licensed vaccines were commercially obtained. Ultrapure LPS (*Salmonella minnesota* R595, List Biological Laboratories, Campbell, CA, USA) was used at 100 ng/ml. Supernatants were assayed by ELISA for TNF (BD Biosciences, San Jose, CA, USA), IL-1β (eBiosciences, San Diego, CA, USA) and by competitive monoclonal enzyme immunoassay (EIA) for PGE_2_ (Cayman Chemical, Ann Arbor, MI, USA). Additionally, assay supernatants were analyzed by magnetic bead multiplex cytokine/chemokine assay (Millipore, Billerica, MA, USA) and analyzed on the Luminex^®^ 100/200™ System employing xPOTENT^®^ software (Luminex, Austin, TX, USA) and Millipore Milliplex Analyst (version 3.5.5.0). PGE_2_ concentrations were determined using the analysis tool at www.myassays.com.

### Flow Cytometry

Monocyte-derived dendritic cells were treated with blocking agent for 10 min (Sigma-Aldrich), transferred to staining buffer [1× PBS, 0.5% (v/v) human serum albumin] and stained for 30 min at 4°C in the dark (1 × 10^5^ cells/staining) with fluorophore-labeled antibodies [CD80/Fluorescein isothiocyanate/Clone L307.4, CD86/phycoerythrin/Clone 2331, CCR7/V450/Clone 150503, and HLA-DR/PerCP-Cy5.5/Clone L243 (BD Biosciences)]. Cells were then centrifuged (500 × *g*, 10 min), washed, fixed [1% (v/v) paraformaldehyde], and acquired using a LSRII flow cytometer employing FACSDiva software (BD Biosciences). Data were analyzed using the FlowJo (Tree Star, Inc., Ashland, OR, USA). Typically, 10,000 events per sample were acquired.

### Production of OMVs and Characterization

Outer membrane vesicles were provided by Janssen Vaccines and Prevention B.V. Briefly, OMVs were produced from *N. meningitidis* H44/76 (B:P1.7,16;F3-3) WT or derivatives. nOMVs were produced from a H44/76 ΔRΔL (15, 16, 27), resulting in a pentaacylated meningococcal LPS from the *lpxL1* mutant *N. meningitidis* strain, lacking the secondary C12:0 acyl chain at the 2′-position of the lipid A. Detergent-extracted OMVs were produced from the WT strain or the ΔRΔL mutant (28). Adsorption to aluminum hydroxide (Sigma-Aldrich) was performed, as described elsewhere (29). Before use, OMVs were diluted in 10 mM Tris pH 7.4/3% (w/v) sucrose to 50 μg total protein/ml. OMV size was determined by dynamic light scattering (DLS) using the Zetasizer nanoseries (Malvern Nano-ZS, l 1/4 532 nm, Westborough, MA, USA).

### *In Vivo* Immunogenicity

Adult Balb/c mice (6–8 weeks, female, 10/group) were immunized with two doses of OMVs (2.5 μg total protein/dose), two doses of Bexsero [at 2.5 μg total protein/dose (equivalent to 1/10th human dose)], or buffer control, subcutaneously in the scruff of the neck. Doses were administered 4 weeks apart, with terminal bleed taken 2 weeks after the second dose. Serum bactericidal assays (SBAs) were performed, as described previously (30), against the WT H44/76 strain with sera from individual mice using baby rabbit sera (Cedarlane, Burlington, VT, USA) as an exogenous complement source. The H44/76 strain was chosen as our OMV formulations were produced from a *N. meningitidis* H44/76 strain and since H44/76 is used as an immunogenicity indicator strain for Bexsero approval. Reported titers are the reciprocal of the serum dilution giving 50% killing. If 50% killing was not achieved the sample was assigned a titer of 4 (half of the lower limit of detection).

### Statistical Analyses and Graphics

Statistical significance and graphs were generated using Prism v. 5.0b (GraphPad Software, La Jolla, CA, USA) and Microsoft Excel (Microsoft Corporation, Redmond, WA, USA). For data analyzed by normalization to control values, column statistics were conducted using the two-tailed Wilcoxon signed-rank test or unpaired Mann–Whitney test as appropriate. Results were considered significant at *p* < 0.05 and indicated as follows: *p < 0.05, **p < 0.01, ***p < 0.001, ^#^*p* < 0.05, ^##^*p* < 0.01, ^###^*p* < 0.001, ^+^*p* < 0.05, ^++^*p* < 0.01, and ^+++^*p* < 0.001.

## Results

### OMVs Incorporating Genetically Attenuated Endotoxin Have Reduced Cytokine Induction Potential in Human Newborn and Adult Blood

**Figure 1:**
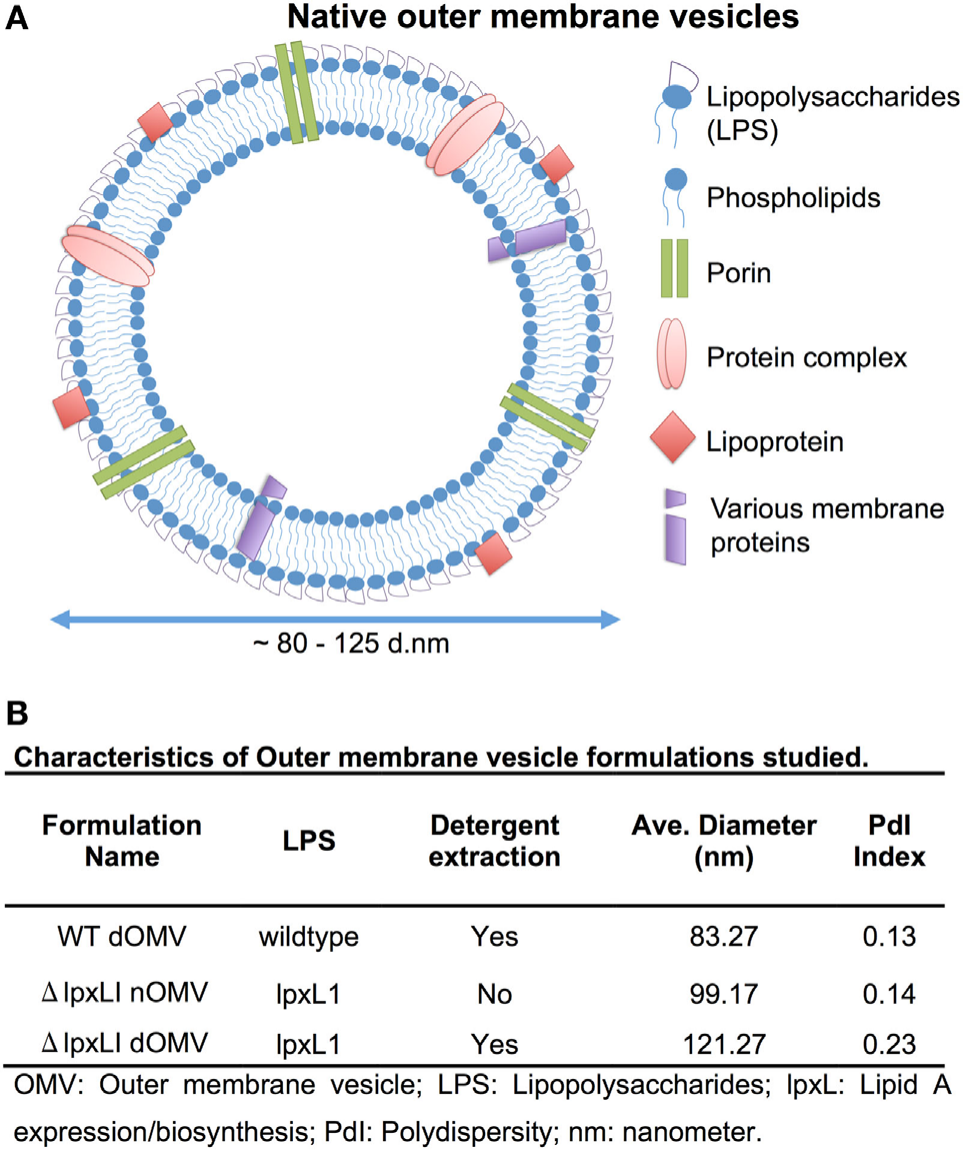
**Depiction and characterization of *Neisseria meningitidis* outer membrane vesicles**. **(A)** Outer membrane vesicles (OMVs) are composed of outer membrane lipids, proteins, and lipopolysaccharide and are produced by the blebbing of membranes of live Gram-negative bacteria during *in vitro* and *in vivo* growth [adapted from Sanders et al. (7)]. **(B)** Characterization of each OMV type used in this study, including LPS type, if detergent extraction was used, quantification of average size in nanometers and polydispersity (PdI) index.

**Figure 2:**
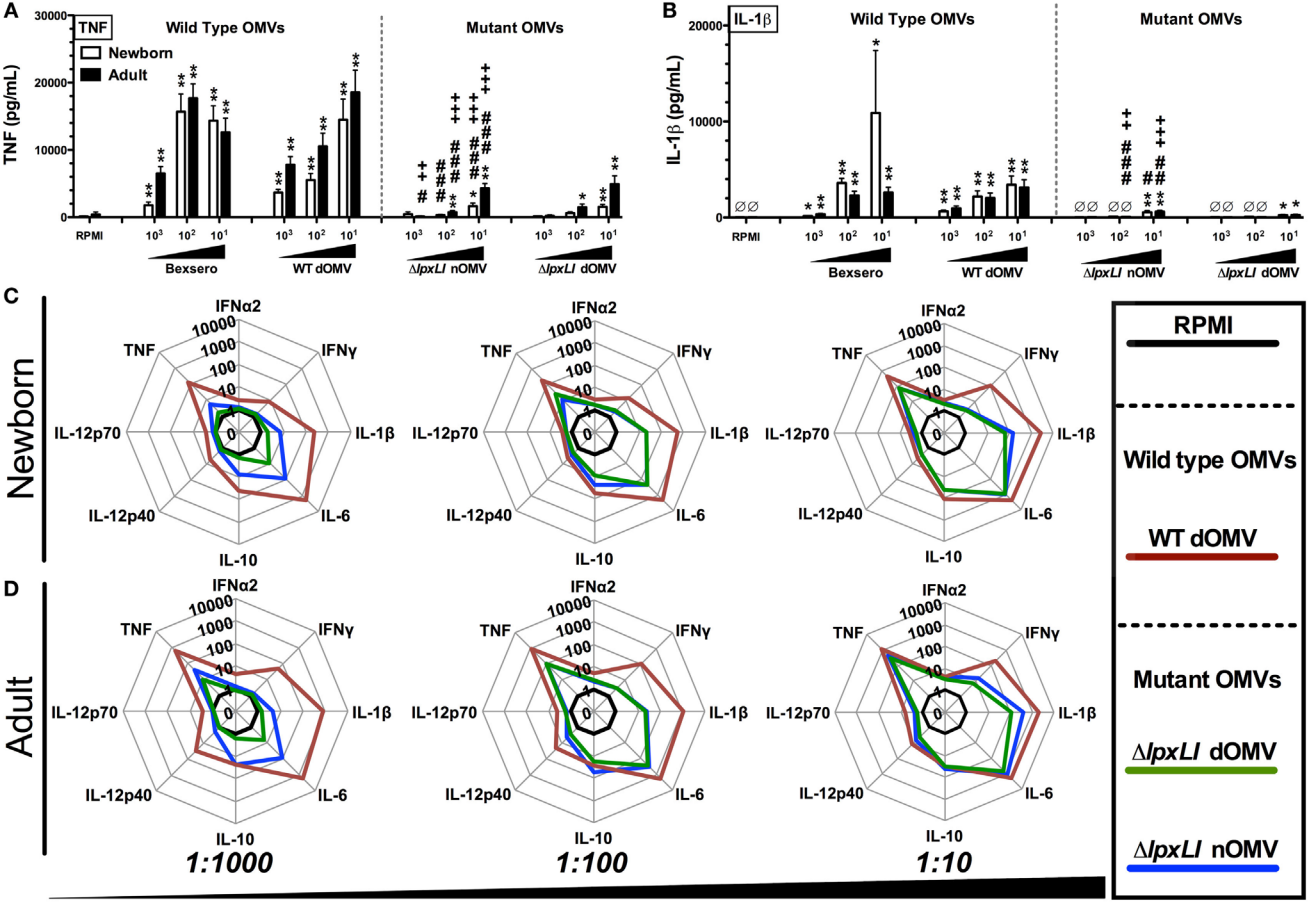
**OMVs containing genetically attenuated endotoxin demonstrate relatively low cytokine induction in human newborn and adult blood**. Human neonatal and adult blood cultured *in vitro* for 6 h with buffer control (RPMI) or with increasing concentrations of wild type (WT) and mutant OMV formulations (1:1000–10 v/v). Supernatants were collected for ELISA **(A,B)** and multiplex assay [**(C)** newborn, **(D)** adult]. Results represent means ± SEM of *N* = 7. For analyses at individual treatments (e.g., control RPMI vs. Bexero 1:10), unpaired Mann–Whitney test was applied at each concentration, and statistical significances are denoted as follows: *p < 0.05, **p < 0.01, and ***p < 0.001. For comparison of Δ*lpxLI* nOMV to Bexsero, ^#^*p* < 0.05, ^##^*p* < 0.01, and ^###^*p* < 0.001. For comparison of Δ*lpxLI* nOMV to WT dOMV, ^+^*p* < 0.05, ^++^*p* < 0.01, and ^+++^*p* < 0.001.

Wild-type OMVs were produced from *N. meningitidis* H44/76 (B:P1.7,16;F3-3), while nOMVs (Figure 1A), incorporating a genetically attenuated version of endotoxin, were produced from a H44/76 ΔRΔL mutant strain (16, 27). All OMVs were characterized by DLS for particle size (Figure 1B; Figure S1 in Supplementary Material), with all formulations falling within a range of 80–120 nm in diameter. Next, we tested the ability of (1) the commercially available Bexsero vaccine, (2) detergent-extracted WT OMV (WT dOMV) (i.e., a Bexsero-like OMV formulation), (3) ΔRΔL mutant nOMV strain (Δ*lpxLI* nOMV) (28), and (4) a Δ*lpxLI* dOMV formulation (Figure 2A), to induce titration-dependent cytokine production in human neonatal and adult blood (Figures 2A,B). Non-detergent treated WT OMVs containing WT LPS were not tested as these are known to be highly cytotoxic. As indicated by measurement of TNF and IL-1β, both Bexsero and the WT dOMV robustly activated neonatal cord and adult peripheral blood in a titration-dependent manner in all concentrations tested, where levels of these two mediators were significantly increased over baseline (*p* < 0.001). The mutant OMVs also significantly induced production of TNF and IL-1β, but only at the highest concentration in newborn blood (*p* < 0.05) (Figures 2A,B; Figure S2 in Supplementary Material). When compared to Bexsero at the highest equivalent volume-to-volume (v/v) dilution tested (1:10), Δ*lpxLI* nOMV demonstrated reduced TNF production in neonatal (*p* < 0.001) and adult (*p* < 0.01) blood. Δ*lpxLI* nOMV were also less effective in inducing IL-1β production in newborn (*p* < 0.05) and WBA (*p* < 0.01). Similar trends were observed when comparing the Δ*lpxLI* nOMV to WT dOMV.

We next broadened our characterization of the ability of WT dOMV, Δ*lpxLI* dOMV, and Δ*lpxLI* nOMV (at v/v 1:1000–1:10) to induce concentration-dependent cytokine production from newborn and WBA using multiplexing assays (Figures 2C,D; Figure S3 in Supplementary Material). When compared head-to-head, the Δ*lpxLI* dOMV and Δ*lpxLI* nOMV induced markedly lower cytokine production profiles as compared to the WT dOMV, especially at the lowest concentration tested. The addition of Alum, the most commonly used adjuvant worldwide, and a component of the Bexsero vaccine to the OMV formulations altered the OMV (both wild type and mutant) induced innate cytokine production profiles (Figures S3 and S4 in Supplementary Material). Most notably, Alum reduced the ability of the WT dOMV to induce IFNγ and the chemokine CXCL10 (interferon inducible 10) (Figures S5A,B in Supplementary Material).

### Δ*lpxLI* nOMV Mature Human Dendritic Cells without Bexsero-Associated Inflammatory Profile

**Figure 3:**
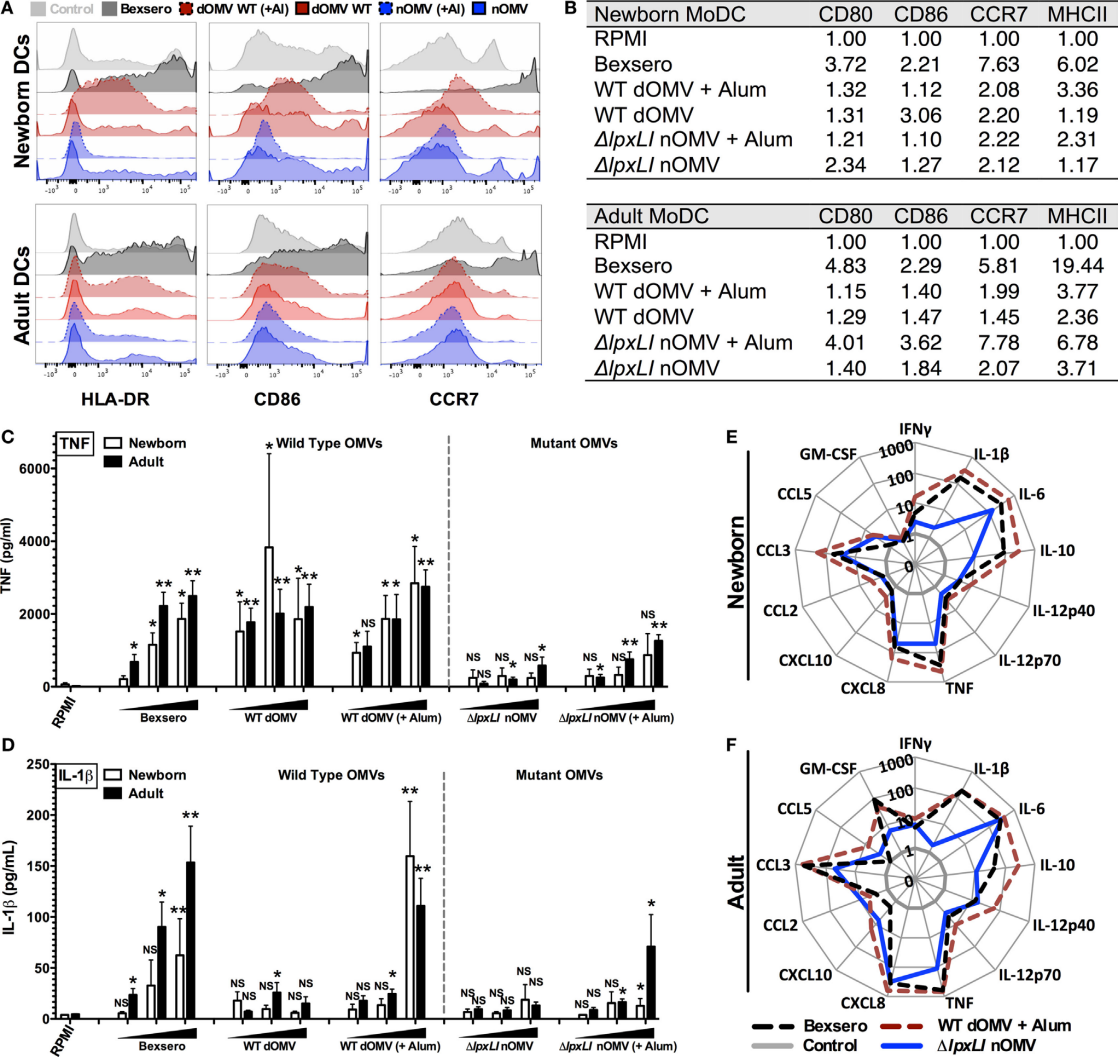
**Δ*lpxLI* nOMV mature human dendritic cells without the high *in vitro* inflammatory profile induced by Bexsero**. **(A)** Human newborn and adult MoDCs were cultured in 10% autologous plasma (v/v) and stimulated for 24 h with OMV vaccines (each at 1:10 v/v). Surface expression of costimulatory molecules and HLA was determined by flow cytometry. **(B)** Average MFI of newborn and adult MoDCs. **(C,D)** MoDCs cultured 24 h with increasing concentrations of OMV vaccines (each at 1:1000, 1:100, and 1:10 v/v). **(E,F)** Multiplex cytokine production from treated DCs. Mean ± SEM, *n* = 5–6. For analyses at individual treatments (e.g., control RPMI vs. Bexsero), unpaired Mann–Whitney test was applied at each concentration, and statistical significances are denoted as follows: *p < 0.05, **p < 0.01, and not significant (NS).

To further characterize the innate immune effects of OMV formulations, 96-well human MoDC-based arrays were generated after culturing CD14 selected monocytes with IL-4 and GM-CSF in the presence of autologous plasma, a rich source of age-specific soluble immunomodulatory factors (19), as described previously (23, 25). In response to Bexsero, a strong upregulation of CD80, HLA-DR (MHCII), and CCR7 and a modest increase in CD86 were observed in both newborn and adult DCs (Figures 3A,B). Both the Δ*lpxLI* nOMV and WT dOMV (with or without Alum) produced slightly reduced DC maturation profiles as compared to the Bexsero. Interestingly, the Alum-adjuvanted Δ*lpxLI* nOMV formulation induced a similar upregulation profile to Bexsero in adult but not newborn MoDCs. Titration-dependent induced MoDC cytokine induction (Figure 3C) supported the flow cytometry results and mostly mirrored those observed in WB. When compared head-to-head, the mutant OMV formulations induced lower cytokine production profiles as compared to the WT formulations, especially at the lowest concentration tested. Interestingly, as seen in the WBA, the addition of Alum selectively enhanced WT dOMV-induced IL-1β production from human DCs (Figure 3D). Moreover, the addition of Alum to the Δ*lpxLI* nOMV formulation only induced a slight increase in IL-1β from newborn (Figure 3D; Figure S6A in Supplementary Material) and adult DCs (Figures 3E,F; Figure S6B in Supplementary Material) at the highest concentration tested (1:10 v/v).

### Δ*lpxLI* nOMV Are Less Inflammatory toward Human Leukocytes than the Majority of Pediatric Vaccines

**Figure 4:**
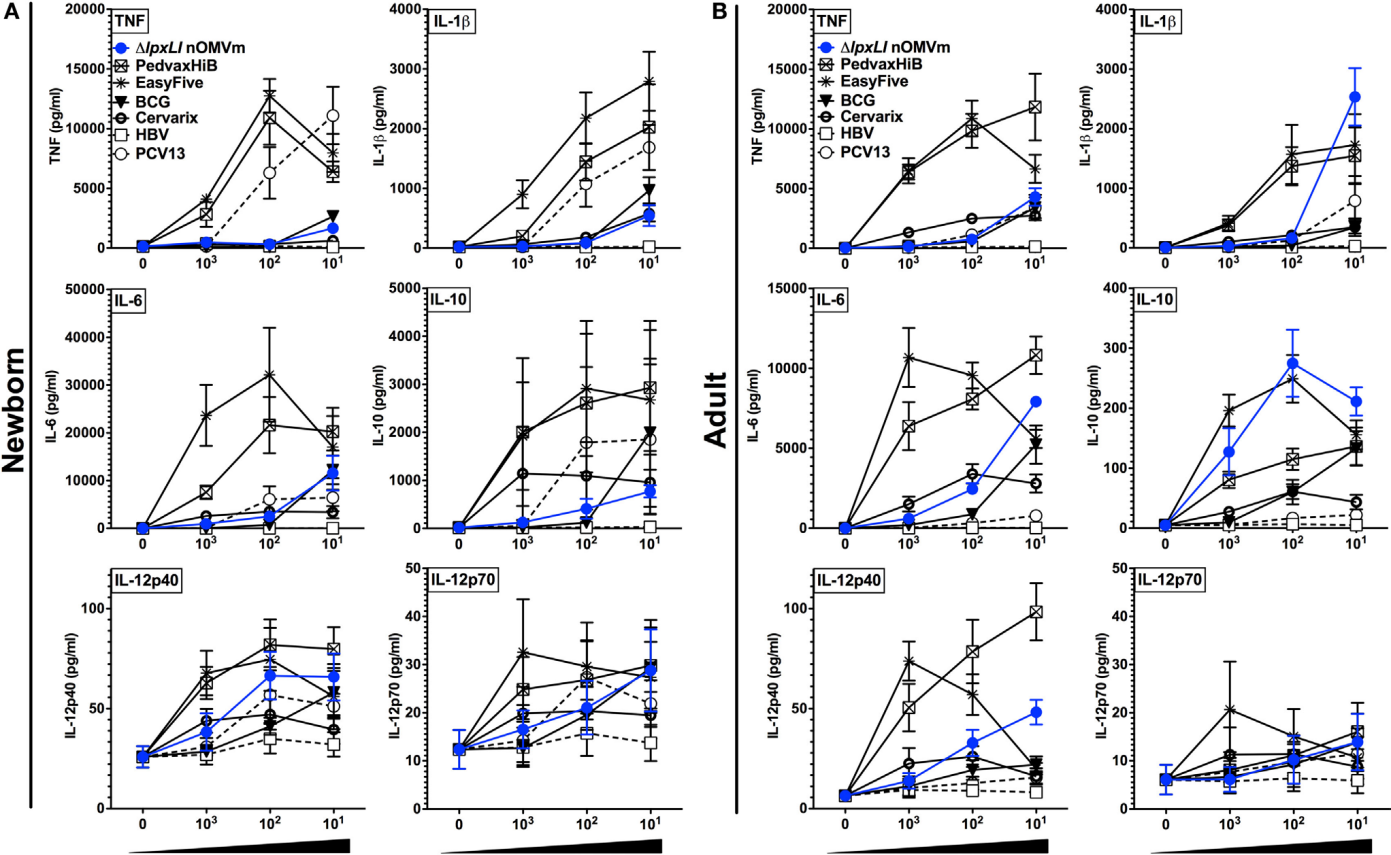
**Δ*lpxLI* nOMV are less inflammatory *in vitro* than licensed combination vaccines**. Human **(A)** neonatal and **(B)** adult blood cultured *in vitro* for 6 h with buffer control (RPMI) or with increasing concentrations of WT and Δ*lpxLI* OMV formulations (each at 1:1000, 1:100, and 1:10 v/v) as well as the vaccines PedvaxHib, EasyFive, BCG, Cervarix, PCV13, and HBV (1:1000–10 v/v). Results for TNF, IL-1β, IL-6, IL-10, IL-12p40, and IL-12p70 are shown and represent means ± SEM of *N* = 7.

To gain further insight into the inflammatory potential of the Δ*lpxLI* nOMV formulation, we next benchmarked it against a number of conventionally licensed pediatric vaccines (Table S1 in Supplementary Material) in both WB and MoDC assays. We have previously demonstrated that several of these licensed vaccines, such as the EasyFive (DTwP-HepB-Hib), induce distinct reactogenicity biomarker profiles *in vitro* (31, 32). When tested at equivalent v/v treatment concentrations, Δ*lpxLI* nOMV conversely induced a lower cytokine response for most innate cytokines (Figures 4A,B) and chemokines (Figure S7 in Supplementary Material) tested, grouping closer to pediatric vaccines such as PCV13 and HBV, than the more inflammatory (Alum + TLR agonist)-containing pediatric vaccines (i.e., PedvaxHIB and EasyFive).

**Figure 5:**
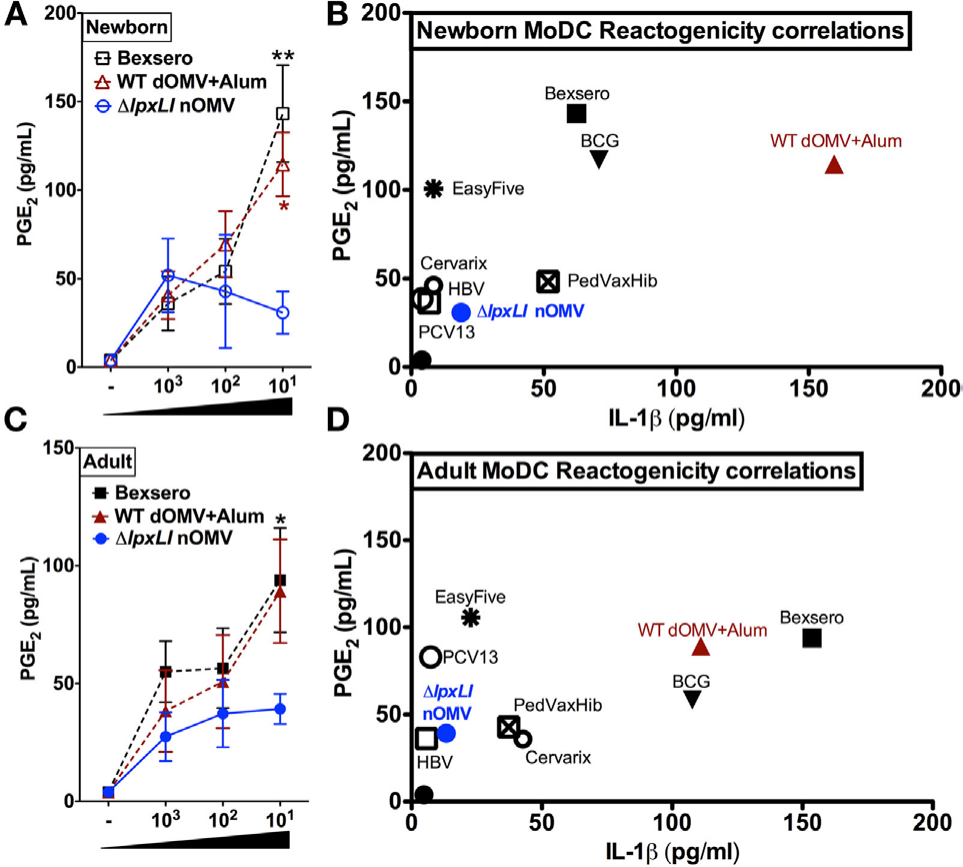
**Δ*lpxLI* nOMV-induced lower MoDC-PGE_2_ and -IL-1β than Alum-adjuvanted WT OMV and the licensed Bexsero vaccine**. Human newborn and adult MoDCs were cultured in 10% autologous plasma (v/v) and stimulated for 24 h with increasing concentrations of OMV vaccines (each at 1:1000, 1:100, and 1:10 v/v). **(A)** Newborn and **(C)** adult PGE_2_ production, as measured by ELISA. Potential reactogenicity biomarker correlation of PGE_2_ with IL-1β for newborn **(B)** and adult **(D)** MoDCs after treatment with wild WT and mutant OMV formulations (at 1:10 v/v) as well as the vaccines PedvaxHib, EasyFive, BCG, Cervarix, PCV13, and HBV (at 1:10 v/v). Mean ± SEM, *n* = 5–6. PGE_2_ levels are depicted with untreated basal levels subtracted. For analyses at individual treatments (e.g., Bexsero 1:10 vs. Δ*lpxLI* nOMV 1:10), Wilcoxon test was applied at each concentration and statistical significances are denoted as follows: *p < 0.05 and **p < 0.01.

Next, immature newborn (Figure 5A) and adult DCs (Figure 5B) were assessed for vaccine-induced production of PGE_2_, a molecule whose *in vitro* production has been correlated with reactogenicity *in vivo* (31–33). Of note, newborn DCs demonstrated significantly reduced Δ*lpxLI* nOMV-mediated PGE_2_ responses as compared to both Bexsero (*p* < 0.01) and WT dOMV (with Alum) (*p* < 0.05) (Figure 5A). A similar pattern was observed for adult DCs, but with significance only observed between Bexsero and Δ*lpxLI* nOMV (*p* < 0.01) (Figure 5B).

Newborn (Figure 5C) and adult DCs (Figure 5D) responses to Δ*lpxLI* nOMV were also benchmarked against conventional licensed pediatric vaccines (at 1:10 v/v, Tables S1 and S2 and Figure S8 in Supplementary Material) in the MoDC array. Here, we focused on the correlation of both PGE_2_ and IL-1β production, as co-production of both in human monocytic assays may predict rabbit pyrogenicity (i.e., fever) *in vivo* (33). Overall, a common trend was observed. The bacillus Calmette–Guérin (BCG) vaccine, Bexsero and WT dOMV consistently induced the highest production of both PGE_2_ and IL-1β. Δ*lpxLI* nOMV conversely induced a lower MoDC PGE_2_/IL-1β profile than WT OMV-based vaccines, suggesting similarity to low reactogenicity pediatric vaccines such as PCV13 and HBV (Figures 5C,D).

### Δ*lpxLI* nOMV Dissociates Inflammation from Immunogenicity

**Figure 6:**
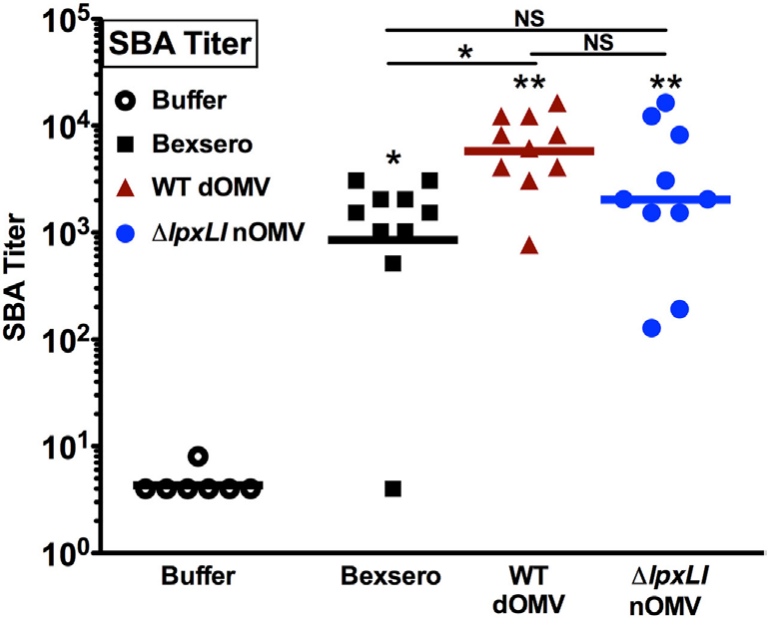
**Δ*lpxLI* nOMV induce robust immunogenicity *in vivo***. Mouse functional antibodies levels were determined by serum bactericidal assays (SBAs) after subcutaneous immunization with Δ*lpxLI* nOMV or WT dOMV (2.5 μg total protein/dose) formulations, as well as the vaccines Bexsero (1/10th human dose) or buffer control. Two doses were administered 4 weeks apart, with terminal bleed taken 2 weeks after the second dose. Mutant OMV demonstrated enhanced immunogenicity over buffer control treatment, and equivalent induction of functional antibodies to the WT formulations, *n* = 10. Horizontal line indicates geometric mean. Wilcoxon test was applied between buffer and each OMV formulation, or between OMV formulations as indicated. Statistical significance is denoted as follows: *p < 0.05, **p < 0.01, and not significant (NS).

Having characterized the relatively low reactogenicity potential of Δ*lpxLI* nOMV toward human leukocytes *in vitro*, we next assessed the ability of Δ*lpxLI* nOMV to induce immunogenicity in mice *in vivo*. Four cohorts of 10 mice each (Figure 6) were immunized subcutaneously with Tris/Sucrose (buffer control), Δ*lpxLI* nOMV or WT dOMV (2.5 μg total protein/dose, dOMVs formulated with Alum), or a 1/10th dose of Bexsero (equivalent to 2.5 μg dOMV). Two doses were administered 4 weeks apart, with blood collected 2 weeks after the second dose to obtain serum for SBA. The buffer control alone failed to induce meningococcal bactericidal antibody responses. In marked contrast, Bexsero (*p* < 0.05), WT dOMV (*p* < 0.01), and Δ*lpxLI* nOMV (*p* < 0.01) all induced robust and significant antibody responses as compared to baseline (Figure 6). Remarkably, even though Δ*lpxLI* nOMVs demonstrated reduced inflammation potential toward human leukocytes *in vitro*, there was no significant difference in immunogenicity as compared to either Bexsero or WT dOMV.

## Discussion

The majority of global immunization schedules are pediatric, with particular focus on newborns and young infants, yet most vaccine discovery programs do not rationally design vaccine formulations for use in humans in early life. Indeed, even when employed, *in vitro* modeling of human responses often occurs in late stage vaccine development. Such current pre-clinical approaches may contribute to vaccine formulations inducing sub-optimal responses in the very young (3). To more completely evaluate an OMV-based *N. meningitidis* vaccine, we took a rational vaccine design approach, in which we combined a relevant *in vivo* model and age-specific human *in vitro* culture systems that together may better model OMV-induced innate immunomodulatory capacity and immunogenicity potential. Overall, when compared to the WT detergent-extracted OMV (WT dOMV), mutant detergent-extracted OMV (Δ*lpxLI* dOMV), and multiple licensed vaccines with respect to innate signaling toward human newborn and adult leukocytes *in vitro*, a prototype Δ*lpxLI* nOMV vaccine generated a pattern of response suggestive of low reactogenicity potential while still demonstrating robust immunogenicity in mice *in vivo*. Demonstration of non-inferior *in vivo* immunogenicity of the Δ*lpxLI* nOMV vaccine, as compared to Bexsero, is noteworthy, especially as the *in vitro* DC maturation profiles are divergent. Such a result may indicate that the inclusion of native endotoxin molecules into traditionally designed OMV-based vaccines may not be as essential for immunogenicity as often assumed.

A key concern regarding modern vaccine development is reactogenicity (33), the propensity of a formulation to cause acute inflammatory events either locally, such as erythema or tenderness at the injection site, or systemically, such as fever. In this context, *in vitro* assays that provide potential reactogenicity biomarker data may be highly advantageous in de-risking formulation selection for *in vivo* use (21). By employing an *in vitro* human DC array, we benchmarked the immunomodulatory abilities of an Δ*lpxLI* nOMV-based vaccine against several licensed pediatric vaccines. DCs are logical targets for such *in vitro* studies as they efficiently process antigens for induction of immunity against pathogens and their components (34). The few published studies that assessed the activating effects of OMV on human leukocytes *in vitro* (35–40) evaluated fixed concentrations with limited cytokine measurements. One study evaluated concentration-dependent OMV responses in human WB and PBMC assays, solely from adult donors (39). Accordingly, our study is the first to comprehensively investigate (a) the ontogeny of OMV-induced immune responses and (b) the responses of human DCs to OMVs as benchmarked against licensed vaccines. Δ*lpxLI* nOMV induced cytokine responses significantly lower than those induced by either Bexsero or licensed vaccines (i.e., PedvaxHIB and EasyFive). As demonstrated by the stronger induction of costimulatory molecule expression by the Δ*lpxLI* nOMV with Alum in adult but not newborn MoDCs, the dramatically enhanced human DC IL-1β production in response to Alum-adjuvanted WT dOMV, as well as our prior studies demonstrating distinct ontological effects of Alum on *in vitro* DC IL-1β production (24), it is reasonable to suggest that our age-specific *in vitro* systems may be useful tools with respect to characterizing candidate vaccine formulations with or without Alum.

Our study has multiple strengths including (a) robust age-specific human *in vitro* modeling, (b) extensive benchmarking to multiple licensed vaccines, and (c) confirmation of immunogenicity *in vivo*. That said, our study also has some limitations including (a) the unavoidable use of different species for *in vitro* (human) and *in vivo* (mouse) studies, (b) that *in vitro* systems, no matter how carefully developed, are imperfect models for responses *in vivo*, (c) the lack of full concordance in the antigens profile found in Bexsero- which contains added recombinant protein- and our Δ*lpxLI* nOMV formulations that do not, and (d) the lack of non-detergent treated- WT strain-derived OMVs that contain WT LPS. While such a formulation was initially not evaluated as part of our current study due to the known cytotoxic effects in humans, based on our results, such WT OMVs may provide a benchmark in future studies employing human *in vitro* systems. Of note, our experiences to date with *in vitro* benchmarking suggest that our *in vitro* studies provide insights relevant *in vivo* (2). Additionally, studies evaluating OMV vaccine-based reactogenicity *in vivo* using similar strategies to ours in mice (41) and humans (42) support these conclusions.

Several aspects of our results will prompt future investigations. For example, nucleotide-binding oligomerization domain (NOD)-like receptor-mediated IL-1β production is greater in human newborn cord than adult peripheral blood (43, 44) and slowly decreases to adult levels over the first years of life (45). A similar phenomenon for basal PGE_2_ levels in WB has been observed (32). Therefore, further study of the IL-1β/PGE_2_ axis as a predictive marker of vaccine reactogenicity may need to take ontogeny into account. Recent use of proteomics (secretomics) has profiled hundreds of proteins released by human monocytes when stimulated *in vitro* with Alum or TLR agonists, several of which have been validated upon study of licensed adjuvanted vaccines, providing opportunities for further refinement of future adjuvanticity and reactogenicity biomarkers (31). Finally, the evaluation of different pentaacyl lipid A mutants (46) and comparison of their modified agonist properties using age-specific human WB and DC assays should also considered.

The persistently high global burden of meningococcal disease in the very young infants and adolescents provides a compelling rationale for developing additional safe and effective early life vaccines (47). Overall, two key aspects of our study deserve particular emphasis: (a) human *in vitro* systems model age-specific biomarker responses that may correspond to reactogenicity and immunogenicity as benchmarked to licensed vaccines and (b) these in *vitro* systems may enable systematic comparison of formulations with and without candidate adjuvants early in the design process to inform translational development. Overall, if further validated, such an approach of *in vitro* assay system-informed age-specific vaccine development may open new paths to more precise vaccine development for distinct vulnerable populations.

## Author Contributions

DD, GD, and OL designed the study. DD, WC, SJ, SB, IB, CP, and SH conducted the *in vitro* experiments. HS, SA, and JF produced OMV vaccines and conducted the *in vivo* experiments. DD wrote the manuscript. OL provided overall mentorship and assisted in writing the manuscript. HS, WC, SJ, SB, IB, CP, SH, SA, JF, and GD contributed to helpful discussions and the careful approval of the final manuscript. All the authors have given final approval for the version submitted for publication.

## Conflict of Interest Statement

HS, SA, JF, and GD are employees of Janssen Vaccines and Prevention B.V., part of the Janssen pharmaceutical companies of Johnson & Johnson. This study was funded by Janssen Vaccines and Prevention B.V. (formerly Crucell B.V.). All the other authors report no potential conflicts.
