# Supplementary material for: A Meningococcal Outer Membrane Vesicle Vaccine Incorporating Genetically Attenuated Endotoxin Dissociates Inflammation from Immunogenicity
Source: Front Immunol. 2016 Dec 8;7:562. doi: 10.3389/fimmu.2016.00562 (PMC5143884; doi:10.3389/fimmu.2016.00562)
Supplement: Supplementary file 1 [file presentation_1.pdf]

Supplementary Material

A Meningococcal Outer Membrane Vesicle Vaccine Incorporating Genetically Attenuated Endotoxin Dissociates Inflammation From Immunogenicity

David J. Dowling[1,2], Holly Sanders[3], Wing Ki Cheng[1,2,4], Sweta Joshi[1,4], Spencer Brightman[1,4], Ilana Bergelson[1], Carlo Pietrasanta[1,2,4,5], Simon D. van Haren[1,2,4], Sandra van Amsterdam[3], Jeffrey Fernandez[6], Germie P.J.M. van den Dobbelsteen[3] and Ofer Levy[1,2,4,*].

[1]Department of Medicine, Division of a Infectious Diseases, Boston Children's Hospital, Boston, MA, USA, [2]Harvard Medical School, Boston, MA, USA, [3]Janssen Vaccines and Prevention B.V. Leiden, Netherlands, [4]Precision Vaccine Program, Division of a Infectious Diseases, Boston Children's Hospital, Boston, MA, USA, [5]Neonatal Intensive Care Unit, Department of Clinical Sciences and Community Health, Fondazione IRCCS Ca' Granda Ospedale Maggiore Policlinico, University of Milan, Milan, Italy, [6]Janssen Research and Development, LLC, Spring House, PA, USA.

*** Correspondence:**
Ofer Levy M.D., Ph.D.

Staff Physician and Director of the Precision Vaccines Program

Division of Infectious Diseases, Boston Children's Hospital

Associate Professor in Pediatrics,

Harvard Medical School,

John F. Enders Research Labs (Room 861.1),

Boston Children's Hospital,

300 Longwood Avenue

Boston, MA 02115

Office: 617-919-2904

Fax: 617-730-0255

E-mail: ofer.levy@childrens.harvard.edu

Keywords: Group B meningococci, Outer Membrane Vesicles, Vaccine, Newborn, Dendritic cells.

1 Supplementary Figures and Tables

1.1 Supplementary Figures

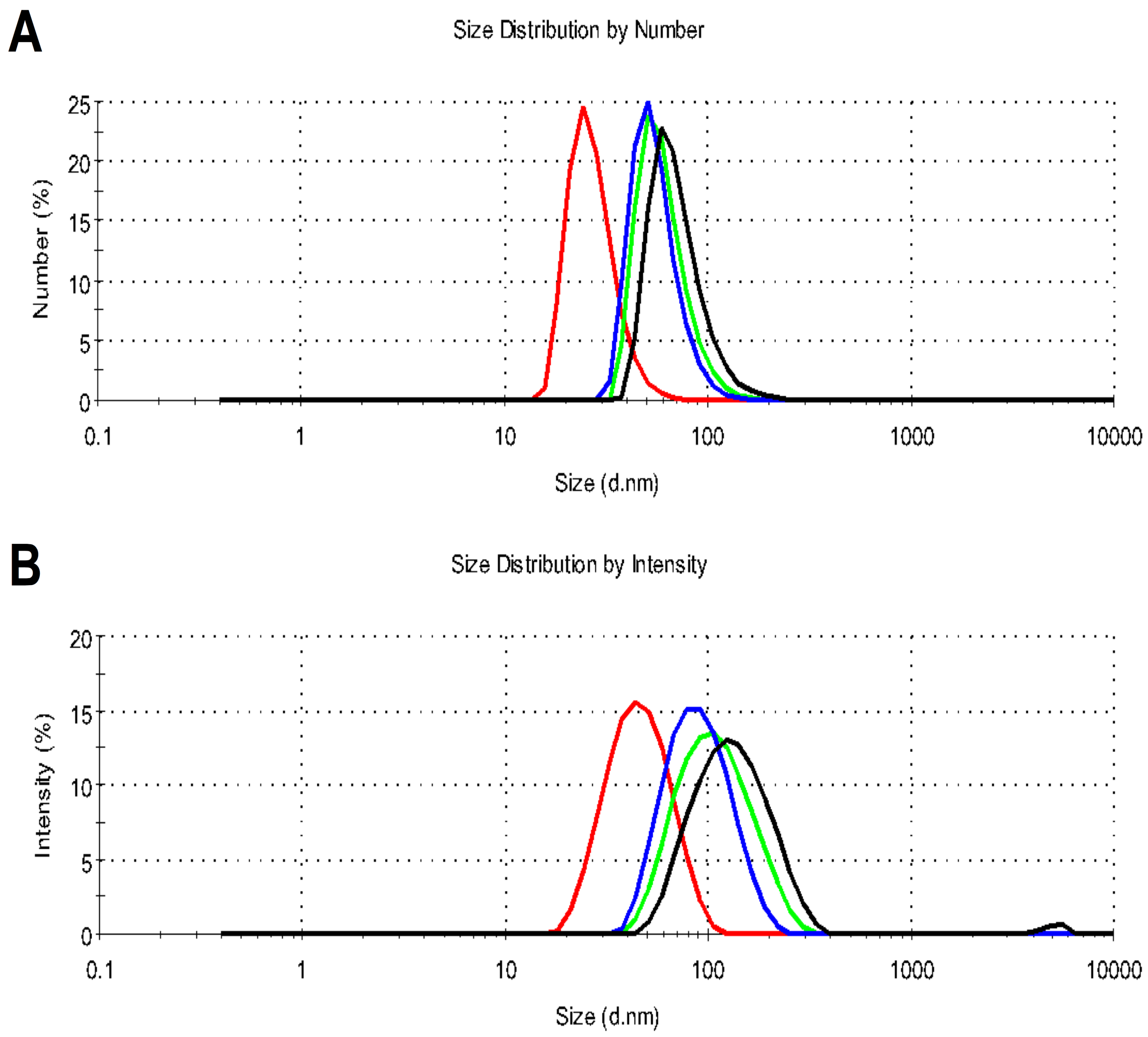

Figure S1. Characterization of OMVs used in this study. Dynamic light scattering (DLS) analysis of OMVs deomonstrating nanometer (nm) size distribution by (A) number and by (B) intensity. WT dOMV are in blue, *ΔlpxLI* dOMV are in black, *ΔlpxLI* nOMV are in green, Control 50 nm beads are in red.

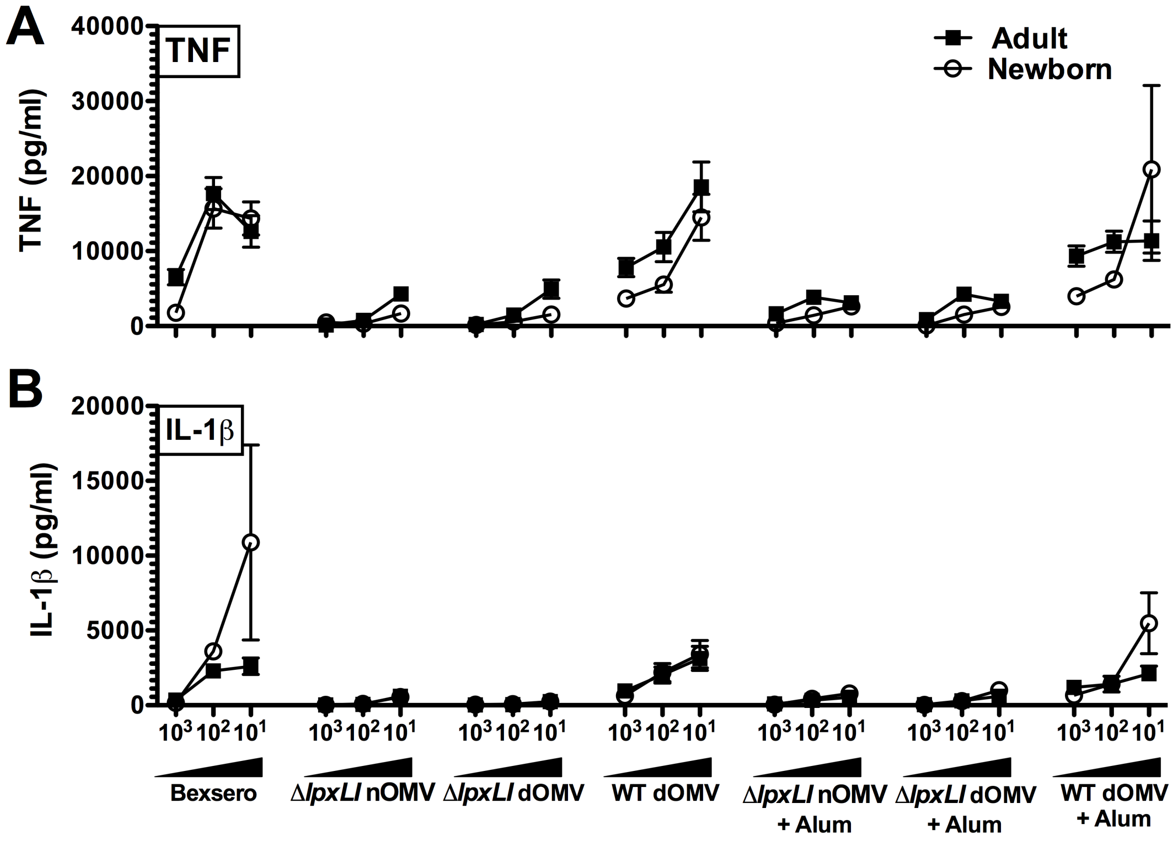

Figure S2. Comparison of OMV cytokine induction potential in human newborn and adult blood. Human neonatal and adult blood cultured *in vitro* for 6 hours with buffer control (RPMI) or with increasing concentrations of WT and *ΔlpxLI* OMV formulations (1:1000 – 10 vol/vol), with or without Alum. Supernatants were collected for TNF (A) and IL-1β (B) ELISA. Results represent means ± SEM of N = 7.

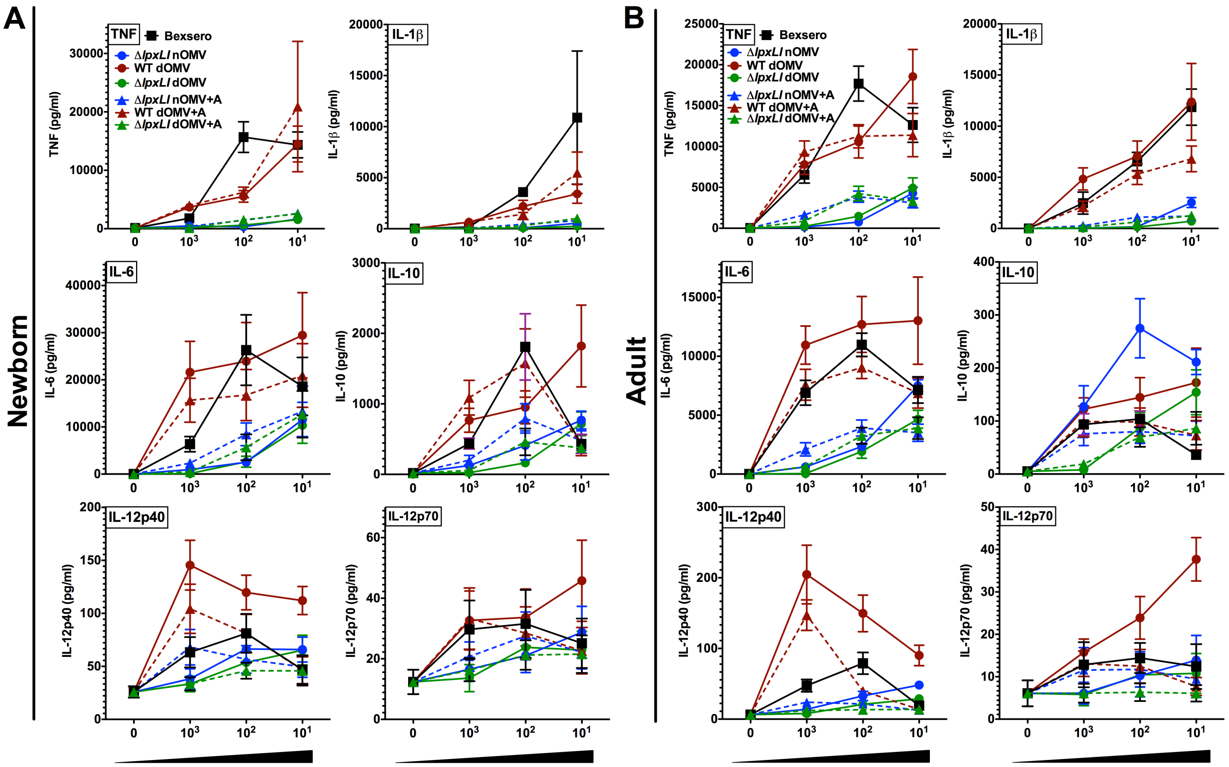

Figure S3. Addition of Alum alters OMV induced innate cytokine production profile. Human (A) neonatal and (B) adult blood cultured *in vitro* for 6 hours with buffer control (RPMI) or with increasing concentrations of WT and *ΔlpxLI* OMV formulations (1:1000 – 10 vol/vol). Results represent means ± SEM of N = 7.

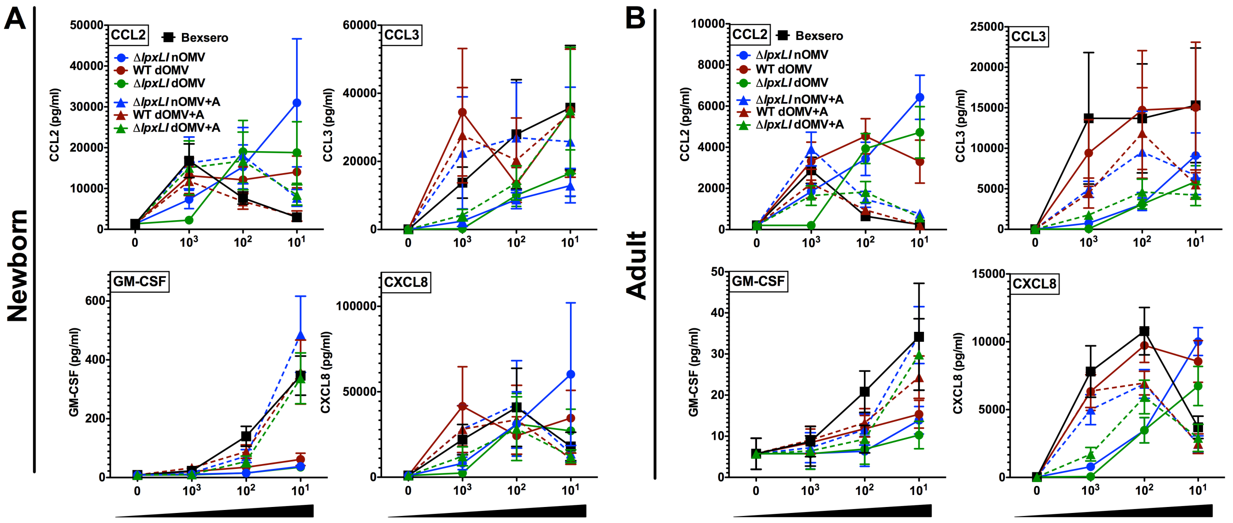

Figure S4. OMV-induced chemokine production profiles in human newborn and adult blood. Human (A) neonatal and (B) adult blood cultured *in vitro* for 6 hours with buffer control (RPMI) or with increasing concentrations of Bexsero or wild type (WT) and mutant OMV formulations (1:1000 – 10 vol/vol). Results represent means ± SEM of N = 7.

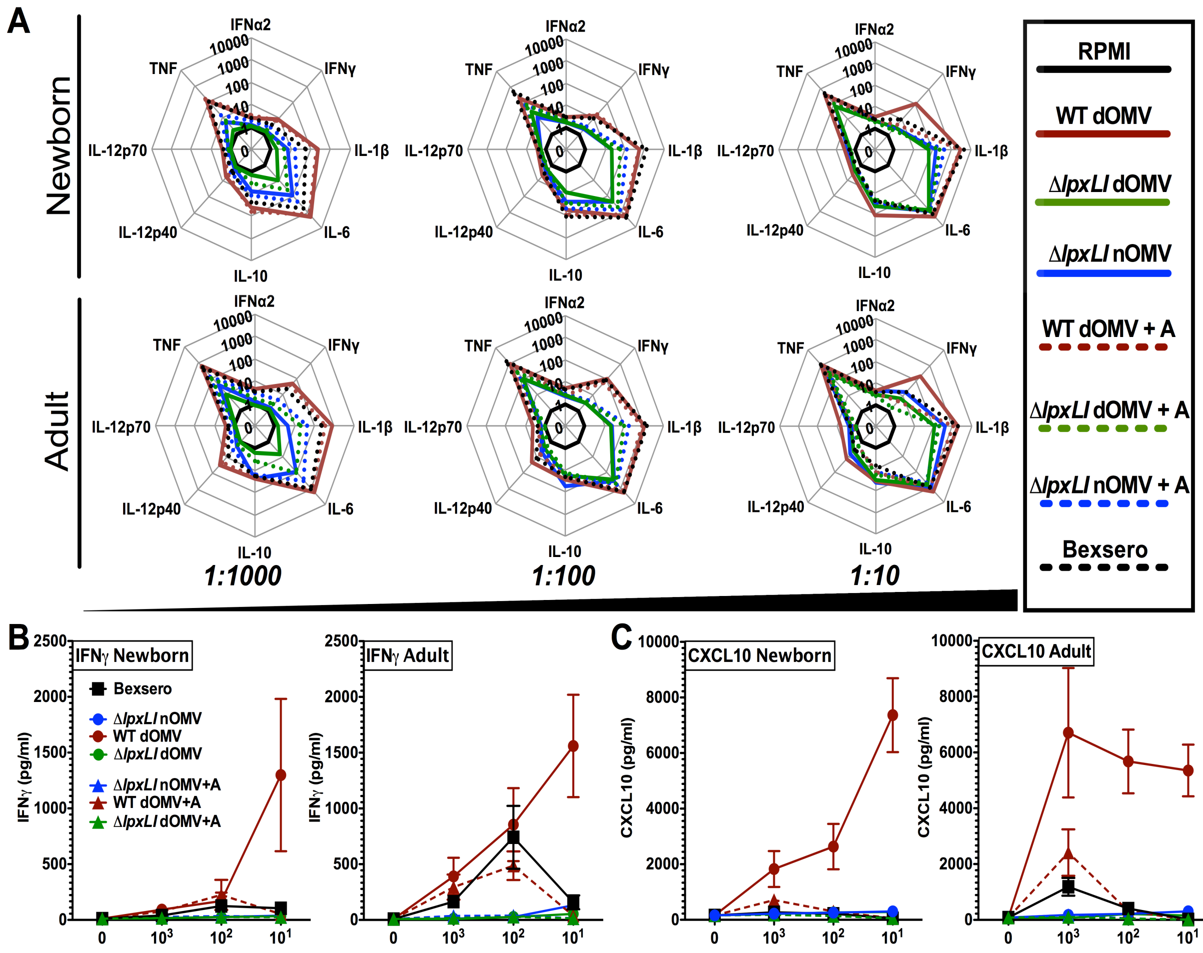

Figure S5. Addition of Alum alters OMV induced innate cytokine production profile. (A) Human neonatal and adult blood cultured *in vitro* for 6 hours with buffer control (RPMI) or increasing concentrations of OMV formulations (1:1000 – 10 vol/vol) with or without Alum. Results represent means ± SEM of N = 7. Relevant OMV formulation without Alum (black), WT dOMV with Alum (red), *ΔlpxLI* dOMV with Alum (blue), *ΔlpxLI* nOMV with Alum (green). Concentration-dependent cytokine production induction was measured in supernatants by multiplex assay. Data are represented as fold-change over vehicle control (black) (mean ± SEM, n = 7). (B) Alum dramatically reduced the ability of the WT dOMV to induce IFNγ and the chemokine CXCL10.

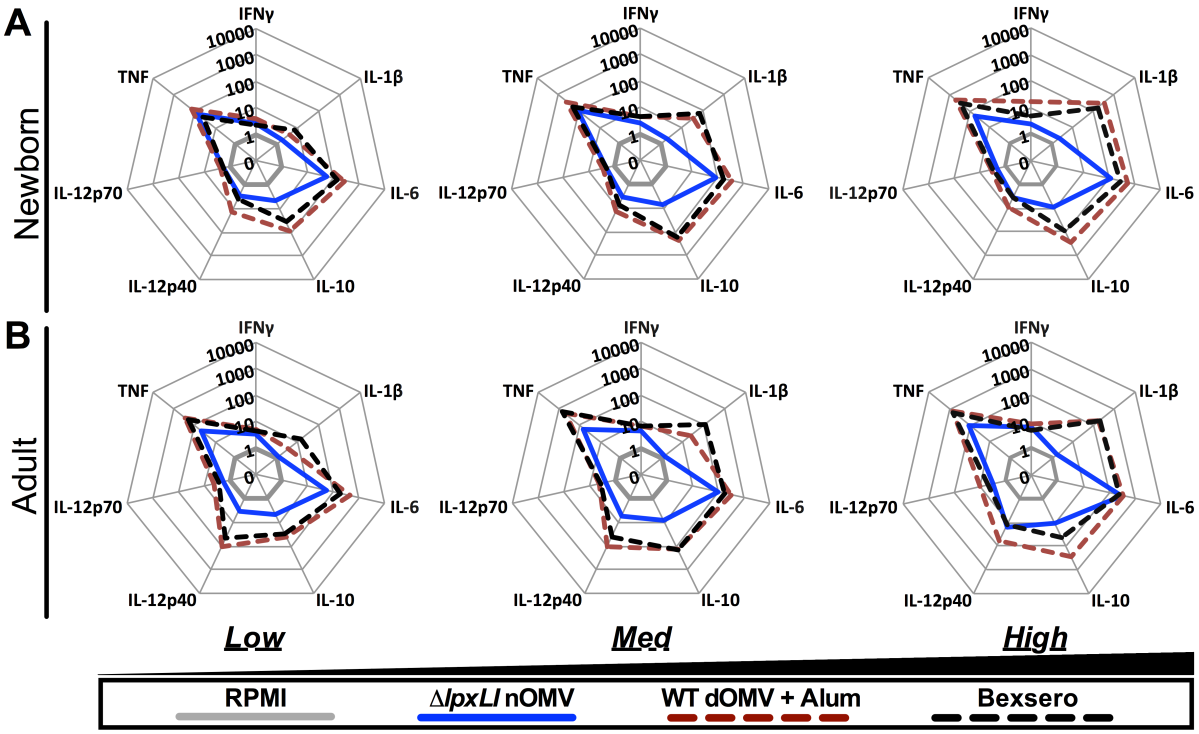

Figure S6. Titration-dependent OMV-induced cytokine production profiles in human newborn and adult MoDCs. Human (A) neonatal and (B) adult MoDCs cultured *in vitro* for 24 hours with buffer control (RPMI) or with increasing concentrations of WT and *ΔlpxLI* OMV formulations (1:1000 – 10 vol/vol). Data are represented as fold-change over vehicle control (gray) (mean ± SEM, n = 5 for newborn and n = 6 for adult).

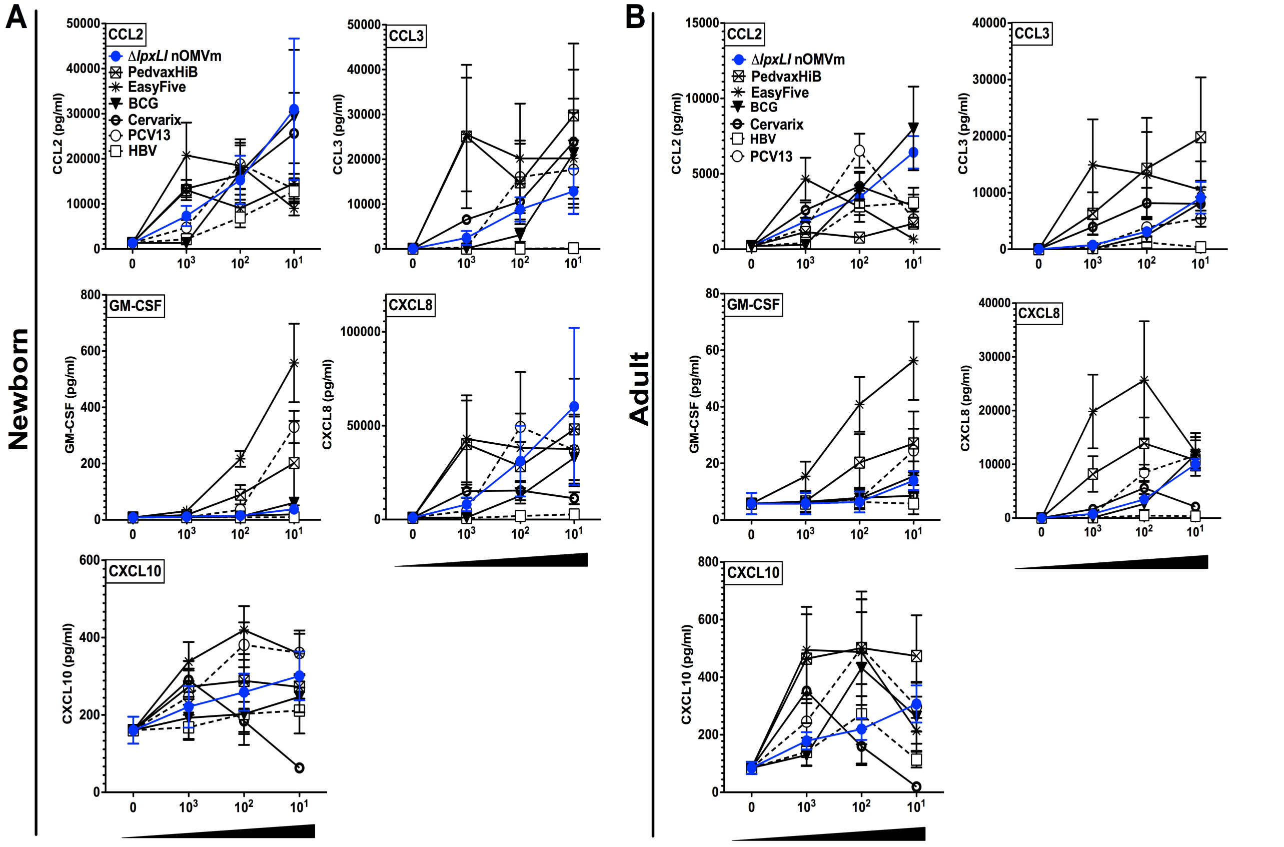

Figure S7. OMV and licensed pediatric vaccine induced chemokine production profiles in human newborn and adult blood. Human (A) neonatal and (B) adult blood cultured *in vitro* for 6 hours with buffer control (RPMI) or with increasing concentrations of WT and *ΔlpxLI* OMV formulations (each at 1:1000, 1:100, 1:10 vol/vol) as well as the vaccines PedvaxHib, EasyFive, BCG, Cervarix,PCV13 and HBV (1:1000 – 10 vol/vol). Results represent means ± SEM of N = 7.

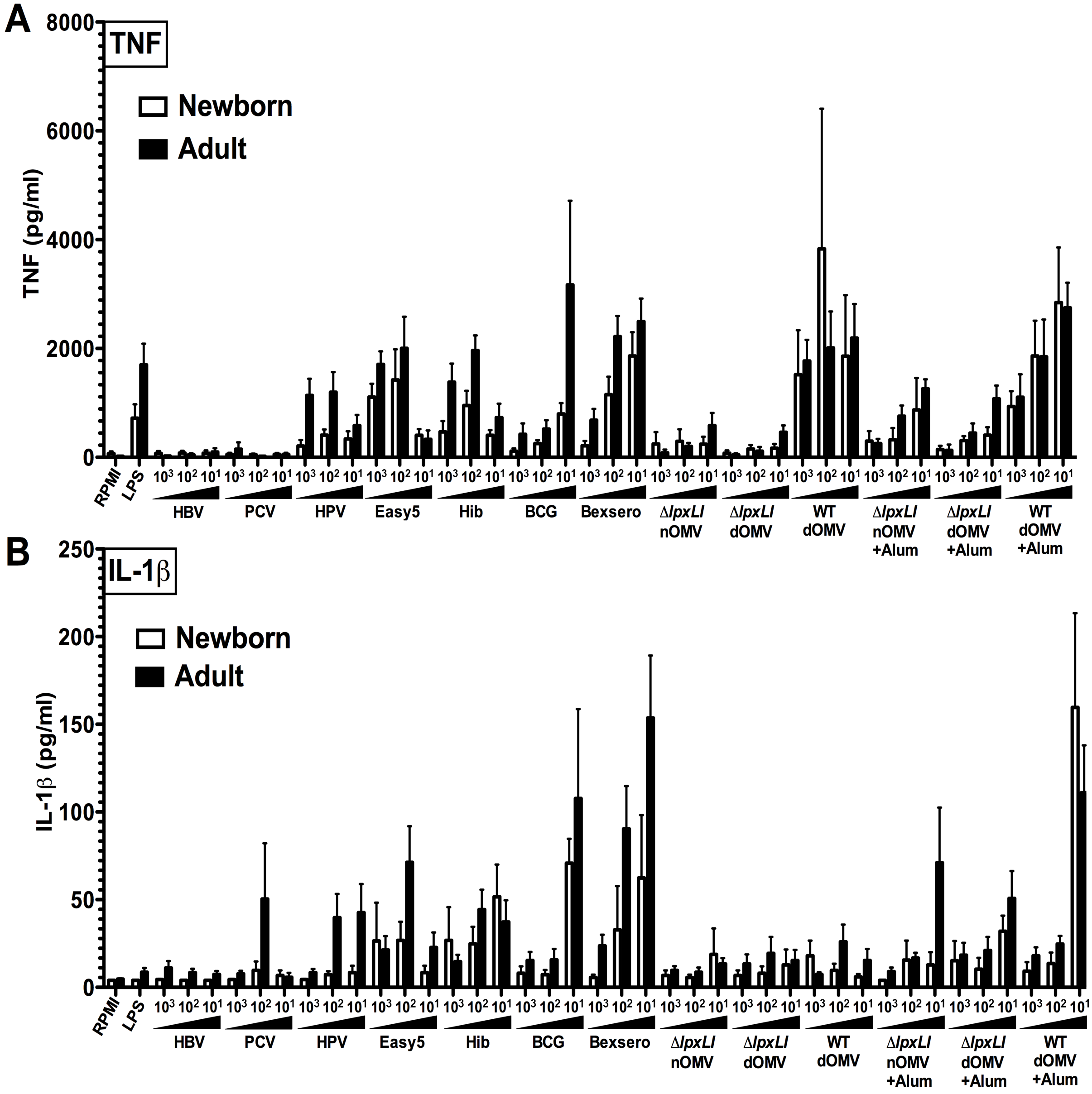

Figure S8. Vaccine-induced TNF and IL-1β production from human newborn and adult DCs. Newborn and adult MoDCs were cultured in 10% autologous plasma (vol/vol) and stimulated for 24 hr with vaccines prior to collection of supernatants for ELISA (mean ± SEM, n = 5 - 6).

1.2 Supplementary Tables

Supplementary Table 1. Vaccines used benchmark OMV induced innate immune responses.

Vaccine	Trade Name	Abbreviation	Manufacturer	Type / Route
Meningococcal	Bexsero	MenB	GlaxoSmithKline	Recombinant Bacterial / IM
Hepatitis B	Recombivax HB	HepB	Merck	Recombinant Viral / IM
Human Papillomavirus	Cervarix	2vHPV	GlaxoSmithKline	Inactivated Viral / IM
Pneumococcal	Prevnar 13	PCV13	Pfizer	Inactivated Bacterial / IM
Tuberculosis	Bacille Calmette-Guerin	BCG	Statens Serum Institut	Live Attenuated / ID
H. influenzae type b, Meningococcal	PedvaxHIB	Hib-MenB	Merck	Combination / IM
Hepatitis B, Diphtheria, Tetanus, Pertussis, H. influenzae type b,	Easyfive	DTwP-Hep B-Hib	Panacea Biotec	Combination / IM

Table adapted from Centers for Disease Control and Prevention Epidemiology and Prevention of Vaccine-Preventable Diseases, 13th Edition. IM; intramuscular, ID: intradermal.

Table S1. Vaccines used to benchmark OMV-induced innate immune responses. Table adapted from Centers for Disease Control and Prevention Epidemiology and Prevention of Vaccine-Preventable Diseases, 13th Edition. IM; intramuscular, ID: intradermal.

Table S2. Vaccine induce human dendritic cells matrtuation profiles. Surface expression of co-stimulatory molecules and HLA was determined by flow cytometry and analyzed as fold-change in mean fluorescent intensity (MFI) vs. vehicle control (mean ± SEM, n = 5-6).

Measurement	Newborn								Adult							
	CD80		CD86		CCR7		MHCII		CD80		CD86		CCR7		MHCII	
	Mean	SD	Mean	SD	Mean	SD	Mean	SD	Mean	SD	Mean	SD	Mean	SD	Mean	SD
RPMI	1.00	0.00	1.00	0.00	1.00	0.00	1.00	0.00	1.00	0.00	1.00	0.00	1.00	0.00	1.00	0.00
LPS (100 ng/ml)	1.58	1.06	2.11	1.71	1.80	0.71	1.42	1.10	1.46	1.11	2.27	2.72	2.19	2.46	4.41	6.99
HBV (1:1000)	1.10	0.38	1.99	2.23	1.58	1.30	1.55	0.59	1.13	0.69	2.40	2.92	1.79	1.47	4.02	5.57
HBV (1:100)	0.80	0.39	2.06	1.53	1.97	1.98	2.43	2.10	1.08	0.48	2.04	2.44	1.78	2.04	3.45	6.11
HBV (1:10)	1.37	1.36	1.80	1.45	1.72	1.06	2.00	1.85	1.54	1.36	1.58	0.87	1.37	0.86	2.15	2.26
PCV13 (1:1000)	1.52	0.73	1.21	0.78	1.28	0.39	1.44	0.78	1.08	0.68	2.15	2.50	1.93	1.98	4.15	6.71
PCV13 (1:100)	1.24	0.64	1.72	1.62	1.55	1.40	1.04	0.49	1.31	0.81	1.60	0.99	1.70	0.84	2.90	3.60
PCV13 (1:10)	1.56	1.30	2.12	2.26	1.86	1.57	1.88	1.92	2.28	2.12	1.54	0.97	1.78	1.12	2.30	2.87
HPV (1:1000)	1.09	1.01	2.29	2.51	2.22	2.16	2.68	3.23	2.66	1.88	1.75	1.95	1.94	1.88	3.80	5.36
HPV (1:100)	1.76	2.42	1.63	1.28	3.74	1.99	4.03	3.32	1.98	1.00	1.74	1.29	2.67	1.82	4.52	4.81
HPV (1:10)	3.87	5.45	2.37	1.55	6.76	2.67	6.82	6.23	5.90	4.41	3.48	4.29	8.27	9.35	13.38	14.41
EasyFive (1:1000)	1.23	0.23	2.06	3.07	1.77	1.25	2.46	3.66	2.69	2.44	1.31	0.68	1.30	0.49	1.53	1.34
EasyFive (1:100)	1.66	1.09	1.65	1.98	2.45	2.30	2.63	4.00	1.71	0.83	1.27	0.66	2.00	1.22	2.06	3.83
EasyFive (1:10)	1.52	1.29	1.49	1.51	1.83	1.81	5.17	7.20	1.88	1.43	0.91	0.45	1.96	1.07	2.43	2.03
PedvaxHIB (1:1000)	1.33	0.59	0.97	0.41	1.27	0.75	1.39	1.38	1.40	1.24	1.11	0.39	1.06	0.33	1.33	0.96
PedvaxHIB (1:100)	2.03	1.98	1.62	1.47	1.91	1.37	1.97	2.06	1.55	0.62	1.69	1.75	1.87	1.67	3.76	5.62
PedvaxHIB (1:10)	2.18	1.67	0.98	0.31	1.41	1.10	1.20	1.25	2.32	1.55	2.30	2.92	2.24	1.97	3.69	6.62
BCG (1:1000)	1.24	0.67	1.83	1.03	1.83	0.81	1.03	0.56	1.82	1.16	1.32	1.05	1.42	1.35	2.46	4.17
BCG (1:100)	2.69	3.01	4.57	7.20	4.35	4.58	4.74	5.64	1.63	0.68	1.32	0.90	1.64	1.49	2.13	2.40
BCG (1:10)	2.51	1.18	1.31	0.76	1.37	0.63	3.62	5.44	2.64	2.32	1.67	2.05	3.03	5.17	2.56	2.84
Bexsero (1:1000)	1.34	0.84	2.36	3.39	1.85	2.18	1.21	0.73	1.31	0.51	1.16	0.39	1.33	0.49	1.55	0.99
Bexsero (1:100)	2.86	4.69	1.68	0.46	3.54	1.62	2.01	0.82	1.40	1.18	1.70	1.25	2.43	1.42	4.33	4.43
Bexsero (1:10)	3.72	5.11	2.21	1.58	7.63	4.69	6.02	8.52	4.83	4.03	2.29	1.42	5.81	3.18	19.44	30.51
WT dOMV (1:1000)	1.81	2.14	1.94	1.72	2.44	2.28	2.29	1.78	1.70	0.91	1.93	2.21	1.92	1.98	3.97	6.90
WT dOMV (1:100)	1.90	1.74	2.18	1.42	2.04	1.07	1.30	0.82	2.70	1.34	2.24	2.29	1.94	1.72	3.43	5.81
WT dOMV (1:10)	1.31	0.95	3.06	3.98	2.20	1.96	1.19	0.45	1.29	0.48	1.47	1.39	1.45	1.03	2.36	2.88
ΔlpxLI nOMV (1:1000)	1.27	0.33	1.61	1.78	1.64	1.70	2.19	2.59	1.50	0.99	2.01	2.47	1.55	1.23	3.62	4.78
ΔlpxLI nOMV (1:100)	1.03	0.48	1.92	2.23	1.59	1.82	0.88	0.22	1.38	0.56	1.98	2.43	1.93	2.06	4.69	6.51
ΔlpxLI nOMV (1:10)	2.34	2.34	1.27	0.82	2.12	1.81	1.17	0.51	1.40	0.67	1.84	2.09	2.07	2.00	3.71	6.57
ΔlpxLI dOMV (1:1000)	1.19	0.47	1.64	1.82	1.39	1.47	0.77	0.43	0.96	0.34	1.81	1.67	2.00	1.86	4.22	6.82
ΔlpxLI dOMV (1:100)	0.97	0.89	1.64	1.38	1.20	0.79	1.93	0.89	1.70	0.57	1.45	0.74	1.65	1.03	3.08	3.89
ΔlpxLI dOMV (1:10)	1.23	0.42	1.48	1.32	1.37	1.04	2.12	2.27	1.62	0.50	1.23	0.51	1.34	0.82	1.99	1.77
WT dOMV + Alum (1:1000)	1.12	0.39	2.74	4.04	2.28	3.02	2.18	1.59	1.76	0.90	1.68	1.54	1.66	1.36	3.52	5.26
WT dOMV + Alum (1:100)	1.70	2.10	1.40	1.16	1.67	1.20	2.17	2.67	1.51	0.82	1.52	1.29	1.75	1.45	3.10	5.64
WT dOMV + Alum (1:0)	1.32	1.78	1.12	0.88	2.08	1.13	3.36	2.76	1.15	0.79	1.40	1.32	1.99	2.20	3.77	4.49
ΔlpxLI nOMV + Alum (1:1000)	2.10	1.83	4.85	9.01	2.02	2.87	1.34	1.05	1.46	0.44	1.22	0.70	1.10	0.31	1.88	1.18
ΔlpxLI nOMV + Alum (1:100)	1.31	0.78	1.43	1.22	1.57	1.29	2.55	2.88	1.32	0.77	1.78	1.96	2.02	2.26	2.81	4.21
ΔlpxLI nOMV + Alum (1:0)	1.21	0.90	1.10	0.92	2.22	1.80	2.31	2.23	4.01	5.13	3.62	6.43	7.78	15.86	6.78	9.72
ΔlpxLI dOMV + Alum (1:1000)	1.09	0.89	2.98	4.44	3.09	4.49	1.48	0.66	1.13	0.52	2.32	3.00	1.89	1.96	4.46	7.09
ΔlpxLI dOMV + Alum (1:100)	1.83	1.74	2.22	2.76	2.82	1.86	1.72	0.77	1.75	0.54	1.93	1.86	2.02	1.10	4.71	5.59
ΔlpxLI dOMV + Alum (1:0)	4.99	5.76	2.80	1.63	9.15	6.40	5.72	6.50	4.79	4.99	2.42	1.51	4.29	2.81	8.42	9.83

Table S2. Vaccine-induce human dendritic cell maturation profiles. Surface expression of co-stimulatory molecules and HLA was determined by flow cytometry and analyzed as fold-change in mean fluorescent intensity (MFI) vs. vehicle control (mean ± SEM, n = 5-6).
